# Dr. Samuel J. Hayes

**Published:** 1897-09

**Authors:** 


### Obituary.

### DR. SAMUEL J. HAYES.

Died at his home in Pittsburg, Pa., June 10, 1897, Dr. Samuel J. Hayes.

Dr. Hayes was born on a large farm near Johnstown, Pa., June 22, 1833. He entered college when about eighteen years of age, paying his way through a course of study principally by teaching. Subsequently he finished his training with a theological course and served several years in the pastorate, being considered successful both in the denominations of United Brethren and the Baptist. In consequence of a severe bronchial affection he was compelled to turn from his chosen profession, and took up the study of dentistry, which he followed during the remainder of his life, about thirty years. The defects of anæsthetic agents in general and the crude condition of the science itself early attracted his attention, and he thereafter devoted himself to the development of this art. In his numerous writings and lectures before schools and associations, both medical and dental, he advocated and sought to establish the rock-bed principles of the science, and is considered an eminent authority on the subject, his definitions for anæsthesia and asphyxia being so clear and forcible that they are accepted as standard. By his researches, and his invention known as "The Hayes Process of Anæsthesia," a means has been given the professional world of producing a true anæsthesia free from peril to operator and patient. This process is now widely known and used in the United States and, to some extent, in foreign countries.

At the time of his death Dr. Hayes was editor and proprietor of the *Dental and Surgical Microcosm*, a journal devoted to the interests of the dental profession, and fearlessly advocating the principles of the art and science of anæsthesia as they were opened up and established by him. He also had in preparation a book on this subject, which failing health compelled him to defer, and which is not yet completed. Com.

